# RORα: a critical nexus in the crosstalk between cholesterol metabolism and macrophage polarization

**DOI:** 10.3389/fimmu.2026.1831854

**Published:** 2026-05-08

**Authors:** Dengju Li, Guangxian Liu, Xiangdong Wen, Guojiang Zhang, Kaixuan Liu, Lin Yuan, Bingbing Yu, Senbo An

**Affiliations:** 1 Department of Joint Surgery, Shandong Provincial Hospital Affiliated to Shandong First Medical University, Jinan, Shandong, China; 2 Department of Orthopedic, Shandong Provincial Hospital Affiliated to Shandong First Medical University, Jinan, Shandong, China; 3 Department of Joint Surgery, Shandong Provincial Hospital, Shandong University, Jinan, Shandong, China; 4 Department of Trauma Orthopedics, Weifang People's Hospital, Shandong Second Medical University, Weifang, China

**Keywords:** cholesterol metabolism, inflammation, macrophage polarization, nuclear receptor, RORα

## Abstract

Retinoic acid receptor-related orphan receptor-α (RORα), a nuclear receptor transcription factor, is essential for maintaining organismal homeostasis and regulating diverse physio-pathological processes. However, its emerging role as a molecular nexus that integrates cholesterol metabolism with macrophage polarization to promote metabolic inflammation has not been systematically summarized. Cholesterol synthesis, transport, and efflux are critical for macrophage polarization. RORα regulates key components of these metabolic pathways and the associated transcriptional mechanisms driving polarization. Moreover, RORα regulates various other immune cells, such as T cells and microglia. This review aims to elucidate the core mechanisms of RORα in the crosstalk between cholesterol metabolism and inflammation, providing a novel method for therapeutic strategies.

## 1 Introduction

Retinoic acid receptor-related orphan receptor-α (RORα), a member of the orphan nuclear receptor (NR) superfamily, is widely expressed in tissues, including the liver, adipose tissue, and muscle (1–3). As a transcription factor, RORα not only regulates circadian rhythms but also governs fundamental physiological processes, including cholesterol metabolism, inflammatory responses, and immune regulation. The structural features of RORα include highly conserved DNA-binding and ligand-binding domains. It modulates gene transcription by recognizing and binding to specific DNA sequences in ROR response elements (RORE) (4, 5). Owing to its unique ligand recognition properties and multifaceted regulatory functions, RORα serves as a critical molecular nexus linking cholesterol metabolism and inflammation. RORα acts as a key regulator of cholesterol metabolism by modulating the expression of associated enzymes (e.g., Glucose-6-phosphate dehydrogenase, cholesterol 25-hydroxylase, and CYP7B1) and transcription factors, including sterol regulatory element-binding protein 2 (SREBP2) and liver X receptor (LXR) (6, 7). Furthermore, cholesterol and its derivatives (such as 7-oxygenated sterols, 24-hydroxycholesterol, and 25-hydroxycholesterol) serve as endogenous ligands that form transcription initiation complexes, precisely modulating the transcriptional activity of RORα (8–10). Consequently, loss of RORα function results in dysregulated cholesterol and lipid metabolism, musculoskeletal dysfunction, and other pathologies (11). In parallel, RORα participates in modulating the systemic inflammatory state by dampening excessive immune reactions and mitigating metabolic inflammation, thereby ameliorating symptoms of metabolic syndrome (12, 13). RORα regulates immune-inflammatory responses via multiple signaling pathways, such as the nuclear factor kappa b (NF-κB) signaling pathways and the interleukin (IL)-17/IL-23 inflammatory axis (14, 15). It can also influence cellular inflammatory reactions by modulating metabolic processes. RORα-deficient mice exhibit low levels of high-density lipoprotein (HDL), elevated inflammatory factor expression, and a predisposition to atherosclerosis (AS) (16). This provides an important basis for understanding the crucial role of RORα in maintaining cholesterol metabolism and inflammatory responses. This dual regulatory capacity establishes RORα as a central component in a complex regulatory network that balances metabolism and inflammation (17, 18).

RORα exhibits pleiotropic roles in circadian biology, metabolism, and immune regulation (19). Its systemic influence stems from its broad tissue distribution and inter-system regulatory functions (20). RORα, as a core clock regulatory component, directly drives the transcription of Bmal1 by binding to the promoter region of target genes, sustaining the rhythmic amplitude of peripheral tissues (e.g., colon, periodontal tissues, and skeletal muscles) (21, 22). RORα deficiency may interfere with the feedback loops of other clock genes such as *MOP3, PER* and *CRY (*23, 24), leading to a disruption of the circadian rhythm. Emerging evidence positions RORα within a lactate-H3K18la-RORα signaling axis that connects sleep disruption to immune dysregulation, underscoring its role at the intersection of metabolism, circadian output, and inflammation (25). Furthermore, RORα is broadly implicated in diverse metabolic processes, including fatty acid and glucose metabolism (26–28). RORα directly regulates the rhythmic expression of genes related to glucose and lipid metabolism including glucose-6-phosphatase and fatty acid synthase (29, 30). MicroRNA-10a-5p generated from adipose tissue macrophages promoted beige adipogenesis *in vivo* and *in vitro* by targeting RORα (31). Therefore, interventions targeting RORα need pay close attention to tissue specificity, circadian biology, and systemic energy metabolism, avoiding unintended off-target effects such as abnormal immune function, metabolic disorders, or neuroendocrine dysregulation caused by multi-pathway regulation.

Cholesterol metabolism exists in a dynamic equilibrium, and its dysregulation is closely related with pathologies, including cancer, cardiovascular disease, and metabolic syndrome (8, 32, 33). A central pathogenic driver of AS is hyperlipidemia, which can lead to the deposition of cholesterol and its metabolites in arterial walls (34, 35). Macrophages are critical cellular mediators in plaque formation and the associated inflammatory response (36, 37). Modulating macrophage cholesterol metabolism to promote M2 polarization suppresses inflammation and slows plaque progression in AS (38–40). Moreover, cholesterol metabolism disorders in patients with metabolic syndrome markedly exacerbate chronic inflammation and immune dysfunction (41, 42). Majdalawieh et al. demonstrated that sesamin inhibits cholesterol synthesis and absorption by regulating PPARα, PPARγ, LXRα, and SREBP signaling pathways *in vitro*, thereby maintaining macrophage cholesterol homeostasis and exerting anti-hyperlipidemic and anti-AS effects (43).

Cholesterol, an essential component of cell membranes, not only maintains membrane integrity and fluidity but also participates in cellular signaling and metabolic regulation (44, 45). The function of macrophages is intensely regulated by intracellular cholesterol metabolism (46–48). The uptake, synthesis, and efflux of cholesterol all profoundly influence macrophage function (49, 50). As key effector cells in the immune system, macrophages exhibit high plasticity (51, 52). They can be broadly classified into two main phenotypes: pro-inflammatory M1 macrophages and anti-inflammatory, reparative M2 macrophages (53–55). M1 macrophages produce abundant pro-inflammatory factors such as tumor necrosis factor alpha (TNF-α), IL-1β, and inducible nitric oxide synthase, which are involved in the inflammatory response (56, 57). Conversely, M2 macrophages promote tissue repair, immune modulation, and the resolution of inflammation (58). M2 macrophages secrete anti-inflammatory cytokines, including IL-10 and transforming growth factor (TGF)-β, upregulate Arg1 expression, and suppress inflammatory responses (59, 60). Cholesterol and its metabolites directly modulate intracellular signaling pathways, influencing macrophages toward M1 or M2 polarization and thereby regulating inflammation and tissue repair processes (61–63).

As a NR, RORα not only regulates cholesterol synthesis and transport but also modulates metabolic and inflammatory signaling pathways to maintain organismal homeostasis (64–66). Given its critical role in cholesterol dysregulation and inflammatory diseases, RORα is emerging as a promising therapeutic target. This review will systematically summarize the role of RORα in cholesterol metabolism and inflammatory responses, particularly macrophage polarization, detailing the underlying regulatory mechanisms and exploring its clinical application prospects.

## 2 Effects of cholesterol metabolism on macrophage polarization

Cholesterol metabolic homeostasis is a critical regulator of macrophage polarization, profoundly influencing inflammatory responses and disease progression. Cholesterol accumulation promotes M1 macrophage polarization, leading to exacerbated inflammation (67–69). Excessive cholesterol accumulation can form cholesterol crystals in macrophages, which promotes reactive oxygen species (ROS) production, activates the NLRP3 inflammasome, drives M1 polarization, and triggers the release of inflammatory factors (70, 71). Macrophage membrane-encapsulated biomimetic nanoparticles can modulate macrophage function by promoting reverse cholesterol transport, thereby reducing cholesterol deposition and clearing excess ROS (72). Low-density lipoprotein (LDL) is converted into oxidized LDL (oxLDL) by excessive ROS; oxLDL subsequently aggravates the inflammatory response through multiple mechanisms (73, 74). Intracellular cholesterol loading in macrophages enhances the uptake of LDL and oxLDL (75). This process drives macrophages to transition into foam cells, releasing large quantities of pro-inflammatory cytokines (such as IL-6 and TNF-α) that induce M1 macrophage polarization. M2 polarization and cholesterol efflux in macrophages are highly dependent on mitochondria, and oxLDL impairs mitochondrial function in macrophages, promoting the M1 phenotype (76, 77). Furthermore, cholesterol loading induces mitochondrial dysfunction in macrophages, resulting in reduced oxidative phosphorylation capacity and promoting the development of the M1 macrophage inflammatory phenotype (50, 78).

As a key component of lipid rafts, cholesterol-induced lipid raft reorganization promotes macrophage polarization toward the pro-inflammatory phenotype, leading to enhanced inflammatory responses and lipid accumulation (79). Additionally, the lysosome is a critical site for regulating cholesterol metabolism as well as homeostasis; cholesterol accumulation in lysosomes can trigger inflammatory activation in RAW264.7 cells (80). Excess cholesterol disrupts lysosomal function. The activity of lysosomal acid lipase (LIPA) is a key step involved in lipid hydrolysis (81). Lysosomal cholesterol accumulation resulting from reduced LIPA activity activates mTORC1 in inflammatory macrophages, blocking AKT-dependent macrophage polarization and promoting glycolytic metabolism, which sustains the inflammatory phenotype.

Cholesterol accumulation is correlated with macrophage polarization and drives inflammatory progression in various disease models. In spinal cord injury mouse models, cholesterol accumulation obstructs reverse cholesterol transport, resulting in macrophage accumulation and fibrosis (82). In a model of metabolic dysfunction-associated steatohepatitis, liver cholesterol accumulation can suppress DHCR7-PI3K expression, promoting macrophage-mediated inflammatory responses (83). In AS, cholesterol crystals induce metabolic reprogramming in primary human macrophages, driving M1 polarization and augmenting inflammatory responses (84).

Dysregulated cholesterol metabolism not only affects macrophage polarization but also modulates macrophage function by regulating signaling pathways. Cholesterol accumulation can activate TLR4/Myd88, an upstream transcriptional regulator of the classical NF-κB inflammatory signaling pathway, thereby enhancing the inflammatory response (85). Cholesterol accumulation-induced NLRP3 inflammasome activation is also a principal driver of the NF-κB signaling pathway (86, 87). Conversely, cholesterol efflux can suppress inflammatory gene expression through LXR-mediated mechanisms (80). In renal clear cell carcinoma, cholesterol and its metabolites influence macrophage polarization by modulating the IGF1R/PI3K/AKT/mTOR signaling pathway to favor the M2 phenotype, which promotes tumor progression (88). Furthermore, high cholesterol levels increase cell membrane rigidity, which may potentiate the activation of inflammatory signaling pathways such as IGF1R/PI3K/AKT/mTOR.

Promoting the normalization of cholesterol metabolism facilitates macrophage M2 polarization and reduces inflammation. Peroxisome proliferator-activated receptors (PPARs), as transcription factors that regulate cholesterol metabolism, are activated to inhibit the expression of IFN-γ and lipopolysaccharide (LPS)-induced inflammatory genes in macrophages (89). LPS not only triggers M1 polarization but also downregulates the expression of PPARs, LXR, and ABCA1 in macrophages, thereby inhibiting cholesterol efflux (89–91). Macrophages can effectively clear excess cholesterol, suppress inflammatory cytokine production, and promote M2 polarization by activating PPARγ, LXR, and their downstream cholesterol efflux transporters, such as ABCA1 and ABCG1 (92, 93). The endogenous LXR ligand 27-hydroxycholesterol (27HC) activates LXR to induce the expression of ABCA1 and ABCG1 (94–96). Regulating the expression of these transporters is crucial for promoting cholesterol efflux to reduce intracellular cholesterol accumulation, protect cellular function, and support the shift of macrophages toward anti-inflammatory M2 polarization. Abnormal cholesterol metabolism leads to the accumulation of cholesterol, which is the direct driving factor for the formation of foam cells (97, 98). MiR-7683-3p derived from M2 macrophage exosomes promotes cholesterol efflux within vascular smooth muscle cell-derived foam cells by activating the PPARγ-LXRα-ABCG1 signaling pathway (99). Perivascular adipose-derived exosomes also upregulate ABCA1 and ABCG1 expression in macrophages, inhibiting foam cell formation (100). Daeshiho-tang activates the PPARγ signaling pathway to modulate cholesterol metabolism by regulating M2 macrophage polarization in AS (36). Additionally, anti-inflammatory cytokines such as IL-4 enhance ABCA1 and ABCG1 expression to activate macrophage cholesterol efflux mechanisms and reduce cholesterol accumulation, promoting macrophage M2 polarization (75). Cholesterol sulfate promotes anti-inflammatory macrophage polarization by modulating ROS and activating the AMPK-CREB signaling pathway, which helps alleviate inflammation after ischemic stroke (101). Understanding the molecular mechanisms by which cholesterol metabolism regulates macrophage polarization, along with exploring strategies targeting cholesterol metabolism pathways, will offer new theoretical frameworks and therapeutic targets for immunotherapy in cardiovascular diseases, tumors, and metabolic syndrome.

## 3 Role of RORα in cholesterol metabolism

Cholesterol metabolism is a precisely regulated process involving multiple steps such as synthesis, uptake, transport, and excretion (102, 103). Among these, reverse cholesterol transport is a crucial step for removing excess cholesterol and preventing its abnormal accumulation (104, 105). This process is mediated by specific transporter proteins that are regulated by RORα. ABCA1 and ABCG1 play key roles in promoting cholesterol efflux and maintaining intracellular cholesterol homeostasis (106, 107). ABCA1 recruits apolipoprotein A-I (ApoA-I) to facilitate intracellular reverse cholesterol transport, participating in the formation of HDL and preserving cellular cholesterol homeostasis (108, 109). Mamontova et al. demonstrated that staggerer mutant mice, which lack functional RORα, exhibit reduced ApoA-I expression and decreased HDL levels (110). Furthermore, dysfunctional RORα in mice lowered serum and hepatic lipids, which coincided with reduced mRNA expression of ABCA1, ABCG1, and SREBP-1c in the liver (111). RORE have been identified in the promoter regions of the *ABCA1* gene (112).

Reverse cholesterol transport is dependent not only on transporter expression but also on upstream signaling molecules. The synthesis of cholesterol is primarily regulated by the SREBP2 signaling pathway (113, 114). SREBP2 maintains cholesterol homeostasis by sensing cholesterol levels in the endoplasmic reticulum and subsequently regulating the expression of cholesterol synthesis-related genes in the nucleus, such as *HMGCR*, *HMGCS*, *ABCA1*, and *LDLR (*115–117). The SREBP cleavage-activating protein (SCAP)-mediated transfer of SREBP2 from the endoplasmic reticulum to the Golgi apparatus is a critical step in this regulatory process (92, 118, 119). Deletion of SCAP alters cholesterol metabolism, thereby inhibiting cholesterol efflux and inducing macrophage M1 polarization (120, 121). RORα and the SREBP signaling pathway exhibit significant crosstalk. RORα modulates SREBP activity, indirectly influencing the expression levels of its target genes to maintain cellular cholesterol homeostasis (122, 123). SREBP, a downstream signaling molecule of LXR, is also regulated by RORα (124).

RORα exerts multi-level regulatory effects on ABCA1, ABCG1 and SREBP in the cholesterol metabolism network. In AS, ChIP and luciferase reporter assays confirmed that RORα directly binds to the promoter region of RORE in ABCA1 and activates its transcription (112). RORα and SREBP exhibit a functional antagonistic relationship. The activation of RORα inhibits the cholesterol synthesis pathway mediated by SREBP (125). RORα promotes cholesterol efflux by maintaining the expression of ABCA1, thereby inhibiting the activation of SREBP (125, 126). When the expression or function of ABCA1 is inhibited by the absence of p53 triggering abnormal activation of SREBP and promoting the pathways related to cholesterol synthesis (127). The decrease in ABCA1 expression leads to the removal of the negative regulation on SREBP, thereby promoting cholesterol synthesis and inflammatory responses (128). During the cholesterol metabolism process, LXR and SREBP form a dynamic feedback loop. When there is an abundance of cholesterol, oxysterols activate LXR, thereby inhibiting the SREBP2 pathway and reducing cholesterol synthesis (129). Conversely, when cholesterol transport is blocked, it inhibits LXR activation and promotes the expression of SREBP2 target genes (e.g. HMGCR and LDLR) to restore cholesterol homeostasis (130). In liver cells, the absence of RORα leads to disordered PPARγ signaling, which indirectly affects the expression of downstream genes of SREBP (2). Therefore, as a transcriptional inhibitor, RORα suppresses the cholesterol metabolism mediated by SREBP while promoting the cholesterol efflux, forming an interdependent regulatory network.

In the regulatory network of cholesterol metabolism, RORα, LXR, and PPARs are members of the NR (131, 132). While each of them executes its own specific functions, they also display considerable functional overlap and regulatory crosstalk to maintain intracellular cholesterol homeostasis. The activation of these three types of receptors upregulates the expression of key efflux transporters such as ABCA1 and ABCG1, thereby promoting reverse cholesterol transport (133, 134). Although their functions are the same, there are significant differences in their upstream activation mechanisms. Different from RORα, LXR is the core sensor of cholesterol metabolism and directly upregulates the expression of ABCA1, ABCG1, and other genes involved in cholesterol efflux (135, 136). In contrast, although PPARγ can also induce the expression of ABCA1, this effect is mainly accomplished through upregulation of LXR indirectly (137). Liang et al. demonstrated that phospholipase A2 group IIA modulated cholesterol efflux possibly through regulation of PPARγ/LXR-α/ABCA1 in macrophages (138). Liu et al. developed a baicalein-copper network to regulate macrophage polarization, and modulate the PPARγ/LXR-α/ABCA1 pathway to promote cholesterol efflux (139). Essential oil from fructus alpinia zerumbet ameliorates AS by activating PPARγ-LXRα-ABCA1/G1 signaling pathway (140).

Cholesterol metabolism does not operate independently but is deeply integrated into the entire lipid metabolism network (61, 141). LDL plays a key role in cholesterol metabolism, and it is responsible for transporting cholesterol to peripheral tissues (142). This process is not only a crucial part of cholesterol metabolism but also relies on the normal functions of apolipoproteins and lipoprotein receptors in lipid metabolism (143). LDL receptor (LDLR) represents the primary pathway for LDL clearance (144, 145). LDLR activity directly influences plasma LDL levels, and any dysfunction can lead to LDL accumulation and an increased risk of cardiovascular disease (146). RORα plays a crucial role in regulating LDLR expression, thereby affecting cholesterol levels (147, 148). Zhang et al. indicated that bergenin can modulate RORα expression, inhibiting abnormal LDLR expression and regulating the expression of genes involved in lipid synthesis and cholesterol metabolism (122). Furthermore, RORα facilitates the hydrolysis and clearance of cholesterol esters by regulating NCEH1 expression, which helps reduce lipid formation in macrophages (26).

## 4 RORα-mediated regulation of macrophage polarization

RORα acts as a dual regulator in macrophage polarization, suppressing the M1 polarization while promoting M2 polarization. RORα modulates macrophage polarization and function through multiple pathways in this process. Cholesterol sulfate, as a RORα agonist, plays a role in promoting macrophage M2 marker expression by inducing STAT6 phosphorylation in RAW264.7 cells, whereas this effect is blocked by the RORα antagonist SR1001 (149). This confirms the dual regulatory function of RORα in inflammatory control. Particulate matter induces nasal epithelial cells to secrete exosomes containing miRNA-19a and miRNA-614 in a respiratory inflammation model induced by air pollution (150). These miRNAs directly target the 3’-untranslated region of RORα mRNA to inhibit RORα expression, promoting the shift of macrophages toward M1 polarization and resulting in the upregulation of inflammatory cytokines and an enhanced inflammatory response. In the nonalcoholic steatohepatitis model, RORα works synergistically with the transcription factor KLF4 to promote M2 polarization mitigating liver inflammation and fibrosis (151). Nobiletin, as a RORα agonist, enhances KLF4 expression, increases the M2 macrophage ratio, and simultaneously suppresses M1 polarization in macrophages along with the expression of inflammatory cytokines (152). In the liver injury model induced by trichloroethylene, the increase in RORα expression can inhibit the M1 polarization of Kupffer cells and significantly reduce the secretion of pro-inflammatory factors, and specific RORα agonist SR1078 effectively inhibits M1 polarization and alleviates liver injury (153). The UBR5/RORα/SPLUNC1 signaling axis activates the GPR132 signaling pathway, driving tumor-associated macrophages to M2 polarization and promoting the formation of a tumor microenvironment (154).

RORα modulates macrophage polarization via several signaling pathways, thereby regulating systemic immune and inflammatory responses. Activation of the NF-κB signaling pathway is pivotal for M1 polarization and inflammatory factor production within macrophages (155–157). RORα is closely involved in regulating the nuclear translocation step of this pathway (158, 159). Inhibitor of kappa B alpha (IκBα) is the primary inhibitor of NF-κB signaling (160–162). It binds to the p65 and p50 subunits to inhibit their nuclear translocation, suppressing NF-κB pathway activation and preventing the initiation of inflammatory gene transcription in the nucleus (160, 163). The overexpression of RORα negatively regulates the NF-κB pathway by binding to RORE in the IκBα promoter, thereby increasing its transcription to inhibit p65 nuclear translocation (164, 165). In acute-on-chronic liver failure, RORα is a target protein regulated by IGF2BP3, and silencing IGF2BP3 impacts the RORα/NF-κB axis and M1 macrophage activation, thereby alleviating inflammation and injury in the liver (166). Han et al. found that RORα prevents LPS-induced macrophage inflammation by downregulating SIRT1 expression and inhibiting the activation of the NF-κB signaling pathway (167). The regulation of RORα in the NF-κB signaling pathway is not limited to macrophages. In adipocytes, RORα upregulates IκBα expression, inhibiting NF-κB activation and the expression of inflammatory cytokines (168). The activation of RORα in intestinal epithelial cells promotes the resolution of colonic inflammation. Activated RORα enhances its association with histone deacetylase 3 (HDAC3), negatively regulating histone H3K9 acetylation levels and thereby suppressing the transcription of NF-κB target genes like IL-1β and TNF-α (169). The downregulation of RORα significantly exacerbates the activation of NF-κB and ERK1/2 signaling pathways in neuronal cells, promoting neuroinflammatory progression in mice with microRNA-7 deficiency-induced neuroinflammation (170).

RORα also indirectly influences polarization by modulating intracellular oxidative stress levels in macrophages. Mao et al. discovered that RORα suppressed the generation of respiratory complex I-dependent ROS by constructing RORα-silenced MCF-10A cells (165). This reduction in ROS mediated the expression of inflammatory cytokines, which inhibited macrophage infiltration and tumor-associated inflammation. Furthermore, melatonin (MLT) activates RORα to inhibit inflammatory factors such as IL-1β, IFN-γ, and TNF-α while simultaneously stimulating antioxidant enzymes and anti-inflammatory factors in patients with systemic lupus erythematosus (171). This protects endothelial cell function, reduces macrophage migration, and effectively alleviates endothelial inflammation.

RORα binding to the RORE directly regulates lipid transporters, metabolic enzymes, and the cholesterol metabolism regulator, thereby remodeling macrophage cholesterol flux and the metabolic microenvironment (172–174). These direct transcriptional regulations result in a significant decrease in the cholesterol content of the plasma membrane, leading to a reorganization of cholesterol-enriched lipid raft domains (175, 176). The activation of TLR4 critically depend on a structurally intact lipid raft platform to facilitate the recruitment of downstream factors (177). By maintaining the expression of ABCA1 and ABCG1, RORα promotes lipid raft disruption, thereby inhibiting the activation of the downstream NF-κB signaling pathway (178–180). Cholesterol efflux prevents the abnormal deposition of free cholesterol on the endoplasmic reticulum (ER) membrane, and protects against NLRP3 inflammasome assembly induced by ER calcium homeostasis imbalance (120, 181). Furthermore, activation of RORα alleviates lipid overload and suppresses mitochondrial ROS generation, resulting in inhibition of the NLRP3 inflammasome activation pathway (182, 183). RORα directly upregulates the transcription of CYP7B1 and SULT2A1, thereby promoting the production of cholesterol sulfate (172, 173, 184, 185). These metabolites act as endogenous agonists of RORα, which activate RORα to create a positive feedback loop exerting anti-inflammatory effects (186, 187).

## 5 RORα-mediated regulation of other inflammatory cells

RORα not only regulates macrophage polarization but also participates in the regulation of multiple immune cells. CD4^+^T cells are the primary cell types expressing RORα, and RORα participates in activating the mTORC1 signaling pathway to promote T cell-induced intestinal inflammation in inflammatory bowel disease (188). Th17 cells represent a subset of CD4^+^ helper T cells that produce inflammatory cytokines such as IL-17 (189, 190). They play a crucial role in the pathogenesis of multiple autoimmune diseases. RORα is indispensable in maintaining the stability and function of Th17 cells (191). Wang et al. found that T-cell-specific RORα deficiency can restrain the progression of autoimmune encephalomyelitis and colitis (192). In autoimmune disease models, T-cell-specific RORα knockout significantly reduces Th17 cell-induced inflammatory cytokine expression, decreases inflammatory cell infiltration, and increases the frequency of regulatory T cells (Tregs) (192). This indicates that RORα is a key regulator in maintaining the Th17/Tregs balance. He et al. demonstrated that the nuclear-transducible form of the RORα transcription modulation domain significantly blocked the potential for naive T cells to differentiate into Th17 cells by competitively inhibiting RORα-mediated transcription in mice with dextran sulfate sodium-induced colitis (193). Increased RORα expression is also correlated with Th17 inflammatory responses in patients with Sjögren’s syndrome. MLT was found to mitigate Th17 inflammation in the peripheral blood of patients by modulating RORα and its related NRs (194). Patrik et al. reported that diosgenin acts as a direct dual-selective inverse agonist for RORα and RORγ, inhibiting their activity in Th17 cells to reduce the expression of IL-17A (195). In CD8^+^ T cells, activated RORα, in conjunction with histone deacetylases, can suppress the NF-κB signaling, maintain cholesterol homeostasis, and regulate immune functions (14, 196). In pulmonary inflammation models, RORα regulates the generation of Th2 cells, and RORα deficiency leads to a significant reduction in Th2 cells (197, 198).

**Figure 1:**
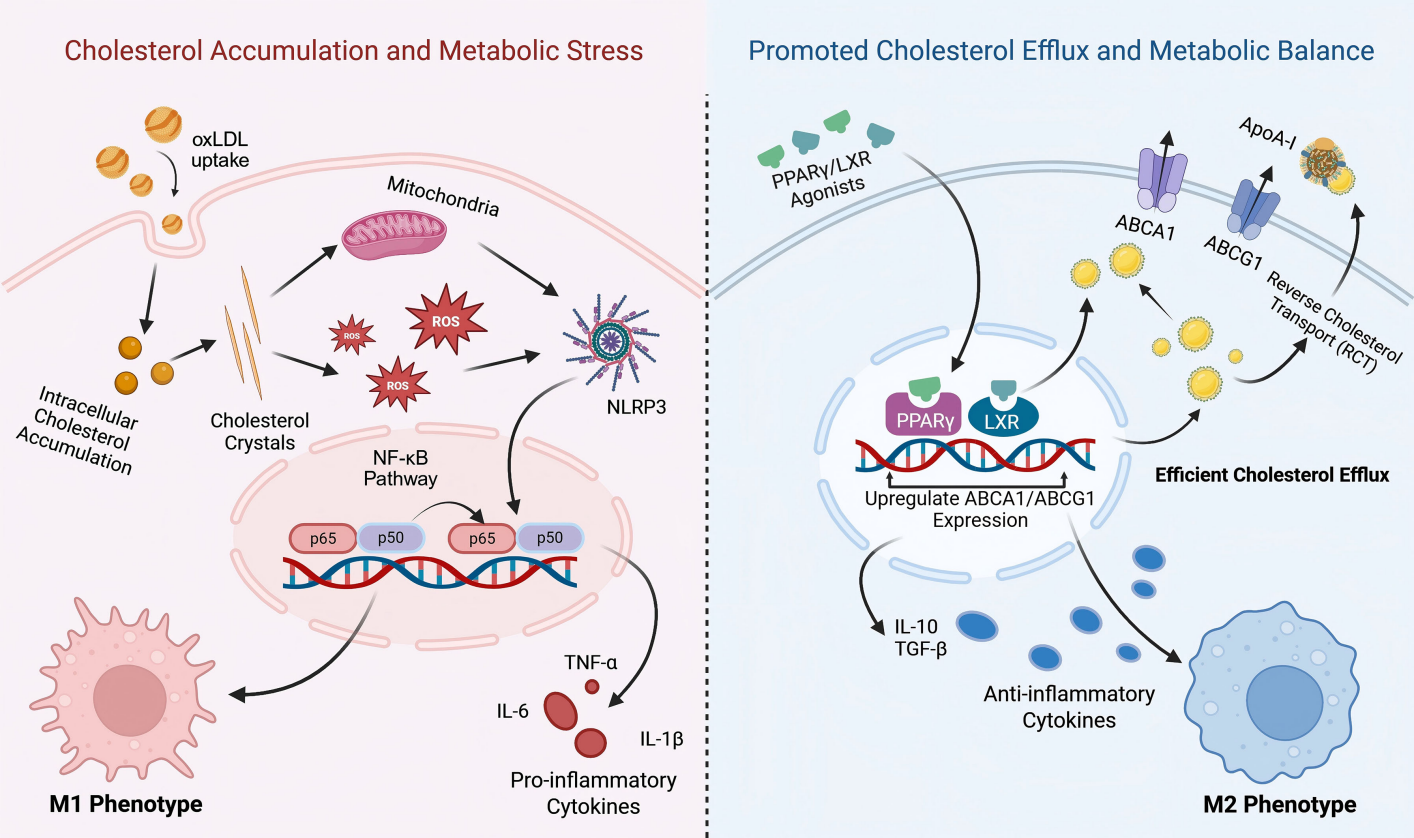
Cholesterol metabolism reprograms macrophage polarization states. Excessive uptake of oxLDL leads to intracellular cholesterol accumulation and crystal formation, which triggers mitochondrial ROS production and NLRP3 inflammasome activation. This metabolic stress sustains M1 polarization via the NF-κB pathway. Conversely, activation of PPARγ/LXR signaling upregulates ABCA1 and ABCG1, promoting reverse cholesterol transport (RCT). Efficient cholesterol efflux relieves metabolic stress, favoring the anti-inflammatory M2 phenotype essential for tissue repair.

**Figure 2:**
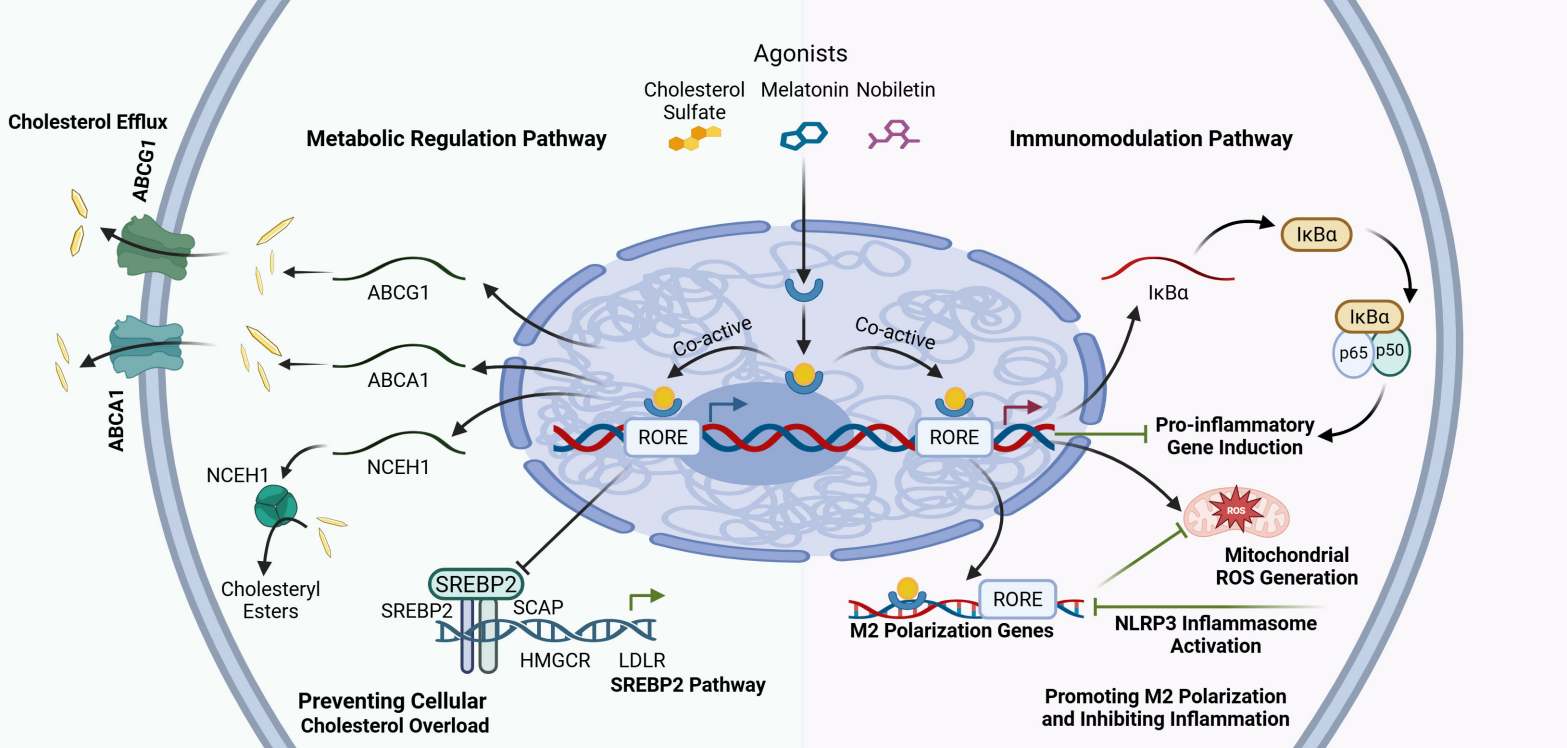
RORα functions as a critical molecular nexus integrating cholesterol homeostasis and inflammatory signaling pathways. Upon activation by endogenous ligands like cholesterol sulfate or exogenous agonists such as melatonin and nobiletin, RORα binds to RORE in the nucleus to orchestrate a dual regulatory program. RORα transcriptionally upregulates the expression of cholesterol efflux transporters ABCA1 and ABCG1, as well as the cholesteryl ester hydrolase NCEH1. Simultaneously, it modulates the SREBP2 pathway to inhibit the expression of lipid synthesis and uptake genes like HMGCR and LDLR, thereby preventing cellular cholesterol overload. RORα directly promotes the transcription of IκBα, which sequesters the NF-κB complex in the cytoplasm, preventing its nuclear translocation and the subsequent induction of pro-inflammatory genes. Furthermore, RORα suppresses mitochondrial ROS generation, which in turn inhibits NLRP3 inflammasome activation.

Recent studies have found that RORα mediates metabolic-epigenetic changes induced by sleep deprivation, thereby regulating the inflammatory response in neutrophils (25). In B lymphocytes, MLT binding to its natural ligand, RORα, can reduce oxidative stress and alleviate inflammatory damage (199). In neuroinflammation models of Parkinson’s disease, MLT exerts neuroprotective effects by modulating RORα to promote M2 polarization of microglia and reduce the expression of inflammatory cytokines (200). Furthermore, MLT activates RORα, inhibiting NLRP3 inflammasome activation to mitigate neuroinflammation and synaptic dysfunction, which offers a new therapeutic avenue for neurodegenerative diseases (201). RORα also inhibits the mtDNA-cGAS-STING-NLRP3 inflammatory signaling pathway in microglia by promoting mitophagy, thereby alleviating neuroinflammatory damage in hypoxic-ischemic encephalopathy and exhibiting significant neuroprotective effects (202). The RORα/γ agonist Tangeretin enhances the expression of the RORα downstream target gene *E4BP4* to inhibit the ERK1/2 cascade and microglial activation, reduce the expression of TNF-α and IL-1β, and improve cognitive impairment in mice (203). RORα overexpression significantly reduces the expression of the inflammatory cytokine IL-33 and the activity of the NLRP3 inflammasome in allergic rhinitis (204). Gao et al. found that serum HDL can reduce the expression of IL-5 and IL-13 in innate lymphoid cells (ILCs) by suppressing RORα (205). ApoE can activate RORα via LDLR and promote the production of inflammatory mediators in ILC2s (206). Furthermore, the number of ILC subsets with upregulated RORα expression increases, accompanied by elevated inflammatory cytokines such as IL-17 (207, 208). These findings highlight the extensive impact of RORα on immune regulation.

Although the macrophage polarization is a key mechanism linking cholesterol metabolism and inflammatory responses, the immune metabolic homeostasis in the tissue microenvironment is not maintained independently by a single cell population (209, 210). The functions executed by RORα in different immune cells can be regarded as a systematic regulatory network. Key components of the cholesterol metabolism network (e.g., HDL and ApoE) are also upstream signals that regulate the activity of RORα in cells such as ILCs (205, 206). RORα maintains the balance between Th17 and Tregs and inhibits the release of inflammatory mediators such as IL-17 (211). This process reshapes the cellular immune microenvironment. RORα regulates cholesterol efflux in CD8^+^ T cells, and this process may alter the lipid composition in microenvironment and thereby affect the metabolic reprogramming of macrophages (196, 212). The improvement of the immune microenvironment mediated by RORα effectively interrupts the cholesterol-inflammation cascade (64). This multi−level regulatory mode further consolidates the central role of RORα in cholesterol metabolism and immune inflammation. A comparison across these cell types reveals a conserved mechanism: RORα consistently acts as a sensor of the cholesterol environment to restrain excessive inflammatory signaling (e.g., NF-κB, NLRP3) and maintain cellular homeostasis. These findings suggest that RORα serves as a universal metabolic checkpoint in the immune system, with its downstream effects finely adapted to the specific tissue microenvironment.

**Figure 3:**
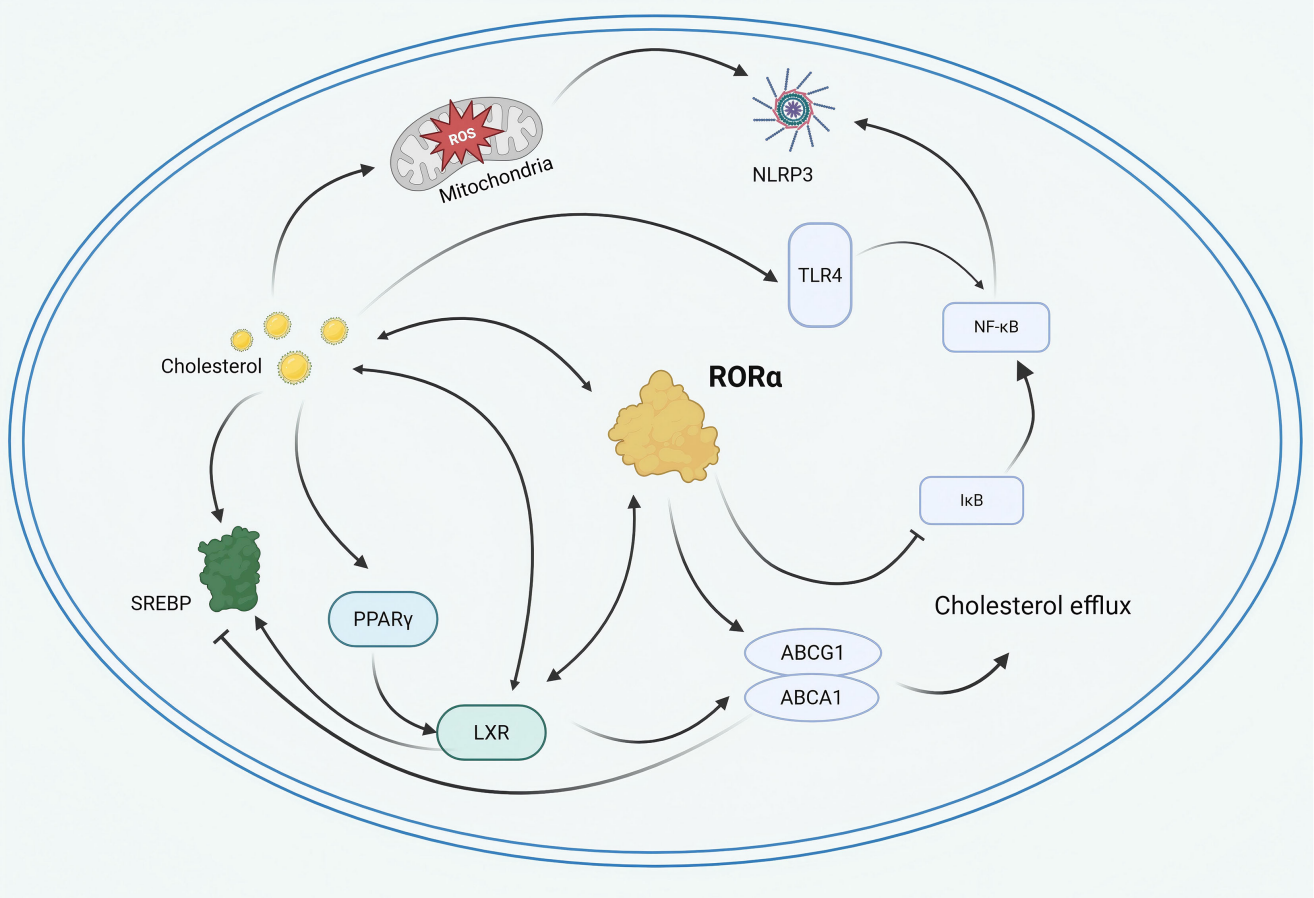
Schematic representation of RORα-centered regulatory loops. RORα cooperates with PPARγ and LXR to transcriptionally upregulate ABCA1 and ABCG1, promoting cholesterol transport. Cholesterol accumulation drives mitochondrial ROS production and NLRP3 inflammasome activation, which in turn triggers NF−κB signaling. RORα regulates this inflammatory response by directly upregulating IκBα.

**Figure 4:**
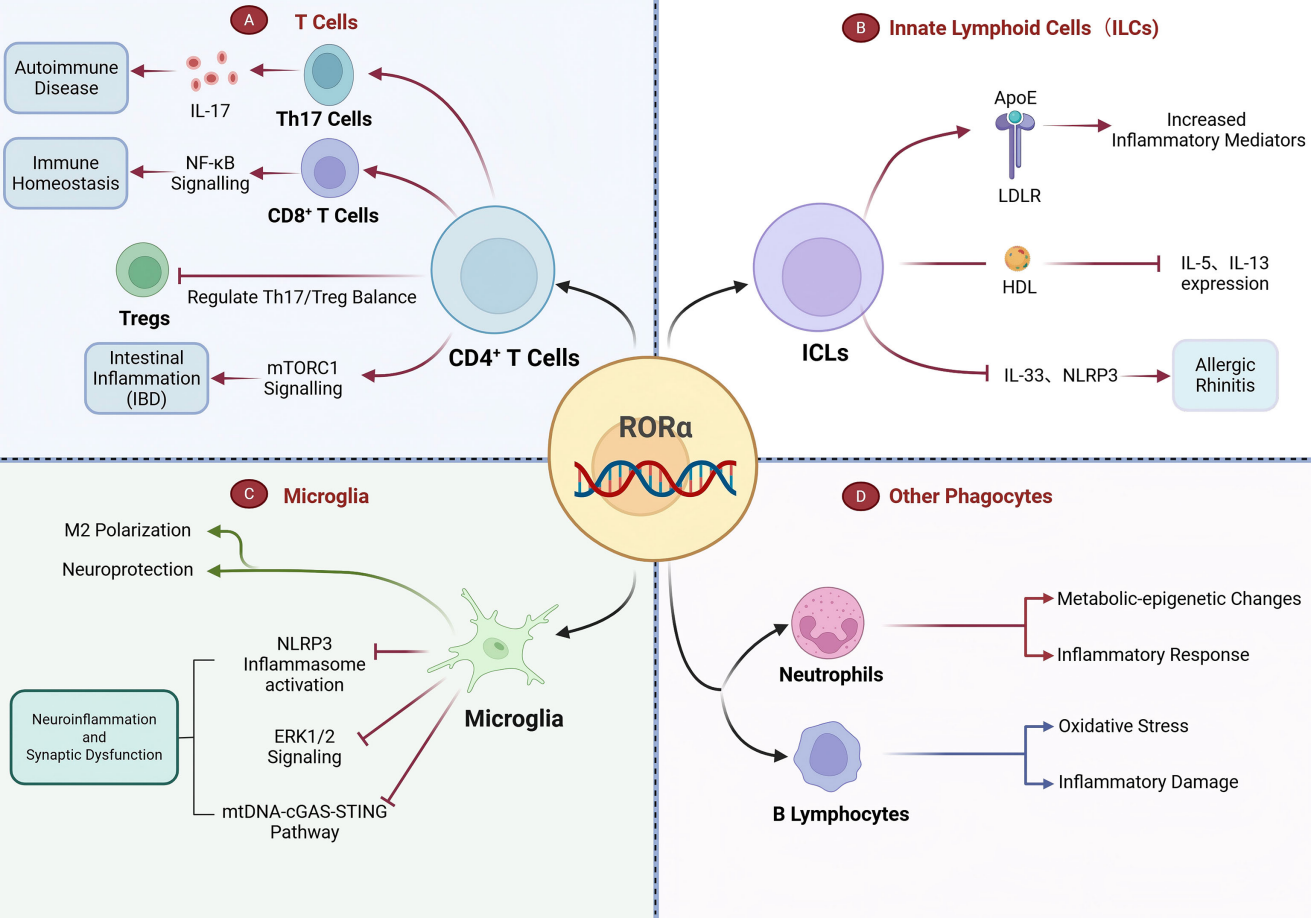
Pleiotropic roles of RORα across diverse immune microenvironments. **(A)** In T cells, RORα is indispensable for maintaining Th17 cell stability and function, regulating the critical balance between Th17 and Tregs in autoimmune diseases. It also modulates intestinal inflammation via mTORC1 signaling in CD4^+^ T cells and maintains immune homeostasis in CD8^+^ T cells by suppressing NF-κB. **(B)** In innate lymphoid cells (ILCs), RORα activity is modulated by lipid metabolism components such as ApoE and HDL to regulate inflammatory mediators in immunity microenvironments. **(C)** In microglia, RORα activation promotes neuroprotective M2 polarization and mitigates neuroinflammation by inhibiting pathways including the NLRP3 inflammasome, mtDNA-cGAS-STING, and ERK1/2 cascade. **(D)** RORα mediates inflammatory responses in neutrophils and reduces oxidative stress in B lymphocytes.

## 6 Conclusions

RORα serves as a critical nexus linking cholesterol metabolism and inflammation, maintaining the delicate balance between metabolic homeostasis and immunity. Through its precise regulation of cholesterol synthesis, transport, and metabolism, RORα not only maintains systemic cholesterol homeostasis but also effectively suppresses inflammatory responses triggered by metabolic imbalance. The regulation of cholesterol metabolism and the functions of immune cells, including macrophages, by RORα involves multi-level and multi-pathway synergistic actions. Elucidating the regulatory mechanisms of RORα in the balance between metabolism and immunity not only provides new perspectives for understanding the pathogenesis of diseases like AS but also establishes an important theoretical foundation for clarifying the mechanisms of cholesterol dysregulation and inflammatory outcomes, such as macrophage polarization.
